# Characterization of relationships between transcriptional units and operon structures in *Bacillus subtilis *and *Escherichia coli*

**DOI:** 10.1186/1471-2164-8-48

**Published:** 2007-02-13

**Authors:** Shujiro Okuda, Shuichi Kawashima, Kazuo Kobayashi, Naotake Ogasawara, Minoru Kanehisa, Susumu Goto

**Affiliations:** 1 Bioinformatics Center, Institute for Chemical Research, Kyoto University, Gokasho, Uji, Kyoto 611-0011, Japan; 2 Human Genome Center, Institute of Medical Science, University of Tokyo, 4-6-1 Shirokanedai, Minato-ku, Tokyo 108-8639, Japan; 3 Graduate School of Biological Sciences, Nara Institute of Science and Technology, 8916-5 Takayama, Ikoma, Nara 630-0101, Japan

## Abstract

**Background:**

Operon structures play an important role in transcriptional regulation in prokaryotes. However, there have been fewer studies on complicated operon structures in which the transcriptional units vary with changing environmental conditions. Information about such complicated operons is helpful for predicting and analyzing operon structures, as well as understanding gene functions and transcriptional regulation.

**Results:**

We systematically analyzed the experimentally verified transcriptional units (TUs) in *Bacillus subtilis *and *Escherichia coli *obtained from ODB and RegulonDB. To understand the relationships between TUs and operons, we defined a new classification system for adjacent gene pairs, divided into three groups according to the level of gene co-regulation: operon pairs (OP) belong to the same TU, sub-operon pairs (SOP) that are at the transcriptional boundaries within an operon, and non-operon pairs (NOP) belonging to different operons. Consequently, we found that the levels of gene co-regulation was correlated to intergenic distances and gene expression levels. Additional analysis revealed that they were also correlated to the levels of conservation across about 200 prokaryotic genomes. Most interestingly, we found that functional associations in SOPs were more observed in the environmental and genetic information processes.

**Conclusion:**

Complicated operon strucutures were correlated with genome organization and gene expression profiles. Such intricately regulated operons allow functional differences depending on environmental conditions. These regulatory mechanisms are helpful in accommodating the variety of changes that happen around the cell. In addition, such differences may play an important role in the evolution of gene order across genomes.

## Background

Genes in prokaryotes are often organized into operon structures. Each operon is a series of genes transcribed in a single mRNA, often identified by the presence of promoters and terminators. It has been reported that genes transcribed in a single operon are functionally related and make up a part of a metabolic pathway [1-3]. Therefore, understanding the operon organization of a genome will lead to better understanding of the functions of genes and the genome.

**Figure 1:**
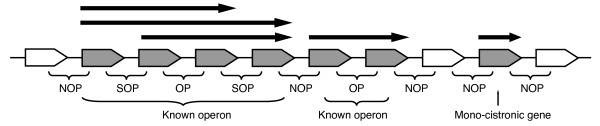
**The schematic model of operon structures**. Arrows indicate the regions transcribed as known TUs. Gray boxes indicate genes in a known operon and open boxes indicate the flanking genes. OP indicates a gene pair in a TU and both genes belong to the same TUs. SOP indicates a gene pair where both genes belong to the same TU and either one of the genes also belongs to a different TU. NOP indicates a gene pair where the genes belong to completely different TUs or, one gene belongs to a known TU and another has not been assigned to any known TUs yet.

Some computational methods have been developed to survey and predict operons [2-20]. To predict operons, gene expression data [5] and co-occurence in functional categories [3,5] have been used. Furthermore, some groups [7,14,20] have predicted operons through a comparative genomic approach. Except for de Hoon *et al*. [10,11,21], which focused on *B. subtilis*, these methods were mainly validated using information from *E. coli*. One of the reasons is that *E. coli *is a well-studied model organism and is characterized by abundant biological knowledge. However, these predictions are not complete and problems still remain in our understanding of the complete details of operon organization. One of the problems for operon prediction is caused by possible fluctuations in an operon's structure, because transcription can occur at different transcriptional units (TUs) depending on the environmental conditions that surround the cell [22-25]. Thus, multiple TUs can be in a single operon. In this case, alternative promoters or terminators are activated by environmental stimuli. In addition, other regulatory mechanisms such as readthrough terminators and riboswitches can also produce alternative TUs in a single operon [26,27]. Therefore, current prediction methods for operon structures are not complete and still need improvement. The terms operon and TU are often confusing because they have such similar meanings. In this study, we use the term 'TU' to refer to a series of genes that are transcribed into one mRNA (an arrow in Figure 1), and 'operon' to refer to a maximal series of genes in which each adjacent pair of genes is contained in at least one common TU (a series of four gray boxes and sixth and seventh gray boxes in Figure 1). To understand such intricate gene transcriptional systems in prokaryotes, a database storing a large number of operons is needed. The availability of RegulonDB [28], a well-established database of operons, regulons and other regulatory elements in *E. coli*, plays a part in the widespread use of this organism in other studies. Since *B. subtilis *also has a long history as a model organism of Gram-positive bacteria [29], its operon organization has also been often analyzed. Information on *B. subtilis *operons has been collected in the Operon DataBase (ODB), that also stored operons obtained from a variety of other genomes for comparative genomics [30]. Therefore, the combination of TUs in ODB and RegulonDB enables us to analyze more of the details of complex operon organization.

In this study, we systematically analyzed the characteristics of operon substructures. We used more than 1000 TUs from these two databases (see Table 1), and we classified adjacent gene pairs into three groups according to the complexity of the operon structures: operon pairs (OP), sub-operon pairs (SOP) and non-operon pairs (NOP) (Figure 1). OPs are always co-transcribed. SOPs are those that cross the boundary of the TUs, as the transcription depends on the presence or absence of internal promoters and internal terminators. NOPs are not co-transcribed. We report here that these differences in operon structures correspond to the distributions of intergenic distances, gene expression profiles and biological pathways. In addition, their conservation across multiple prokaryotic genomes also correlates with the complexity. These results imply that there can be functional differences within an operon depending on the actual transcriptional boundary and that the differences can also influence genome organization. Our work would be helpful to understand the diversity of operons and improve operon predictions.

## Results

### Classification of adjacent gene pairs based on operon structures

We classified adjacent gene pairs located on the same strand into the following three groups (Figure 1): (i) operon pairs (OPs), (ii) sub-operon pairs (SOPs), and (iii) non-operon pairs (NOPs). TUs in an operon can change due to the presence or absence of internal promoters or internal terminators. Therefore, the TUs of an operon are not always unique and other possible TUs may exist. We defined a gene pair as a SOP when both genes belong to the same TU and one of the two genes also belongs to another TU. For example, in Figure 1 the second and third genes from the left, say genes A and B, respectively, comprise a SOP because they belong to the same TU (the longest arrow), and gene A also belongs to another TU (upper arrow) to which gene B does not belong. In the same manner, the forth and fifth genes are also regarded as a SOP in Figure 1. OPs are gene pairs in known operons where both genes belong to the same TU and no other TU. NOPs are defined as either gene pairs where the two genes belong to completely different but known TUs, or where one gene belongs to a known TU and the other has not been assigned to any TUs. Because we define a mono-cistronic gene as a type of TU, gene pairs that include a mono-cistronic gene are always regarded as SOPs or NOPs. Table 2 shows the number of these pairs in *B. subtilis *and *E. coli*.

### Genomic properties of operons

**Figure 2:**
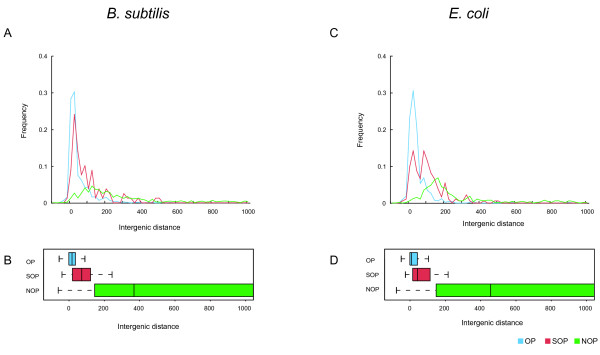
**Distributions of frequency of intergenic distances between adjacent genes**. The upper figures are the distributions of intergenic distances (bp) between adjacent genes at 20 bp intervals in *B. subtilis *(A) and in *E. coli *(C), and the bottom figures are their box plots (B and D). The leftmost, rightmost and middle vertical lines of the boxes represent the first and third quartiles and median value, respectively. The intergenic distances more than 1000 bps are not shown. Blue, red and green lines indicate OPs, SOPs and NOPs, respectively.

It is already known that the intergenic regions between genes within an operon are smaller than those in different operons in the *E. coli *genome [6]. The *B. subtilis *genome also has a similar distribution of intergenic distances [11]. Figure 2 shows the distribution of intergenic distances (bp) and box plots for the three groups of adjacent gene pairs in *B. subtilis *and *E. coli*; negative distances mean that the gene pairs partially overlap. It is clear that the distances in both species show a similar distribution pattern. The distances of almost all OPs and SOPs are short. On the other hand, NOPs have larger intergenic distances (p < 1E-14 for OP-NOP and SOP-NOP, chi-squared test). This result is in agreement with past results that showed that gene pairs within an operon and those between operons are clearly different in *E. coli *[6].

Furthermore, we would like to point out that OPs and SOPs have different distributions (p < 1E-11, chi-squared test) despite the fact that both gene pairs are contained within the same TU at least once. These results are more clearly shown in the median values in Table 2 and in the box plots in Figure 2. For example, the medians of intergenic distances for OPs (17 bp and 9 bp) are smaller than SOPs (72 bp and 54 bp) in *B. subtilis *and *E. coli*, respectively. And both values are much smaller than the values for NOPs (376 bp and 467 bp) in both species (Table 2). A schematic view of these contrasts in median values among OP, SOP and NOP are shown in Figure 2B (*B. subtilis*) and Figure 2D (*E. coli*). Since the distributions were so similar even between distantly related organisms such as *B. subtilis *and *E. coli*, in terms of operon organization, we expect the differences in the intergenic distances to be evolutionarily conserved and similar across a broad range of prokaryotic genomes.

#### Conservation of adjacent gene pairs

**Figure 3:**
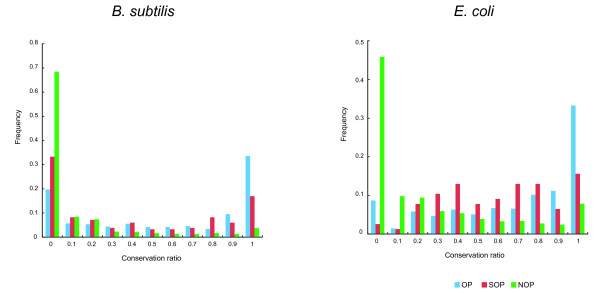
**Conservation of adajcent gene pairs across genomes**. The left figure indicates the conservation ratio based on the gene pairs from *B. subtilis*, and the right figure is from *E. coli*. The ratios of adjacently conserved gene pairs for OPs, SOPs and NOPs are shown in blue, red and green, respectively.

To investigate the relationships between the levels of gene co-regulation in OPs, SOPs and NOPs, and their evolution, we surveyed the conservation of the three groups of adjacent gene pairs among 185 prokaryotic genomes (Fig. 3). We counted the ratio of the gene pairs in *B. subtilis *and *E. coli *that are conserved adjacently in other genomes. The conservation ratio for a gene pair represents the fraction of genomes considered in which the adjacency of the gene pair is conserved. Figure 3 shows the conservation ratio of the gene pairs, where the x axis indicates the ratio and y axis indicates the frequency. When the adjacently located ortholog pairs are conserved across many genomes, the conservation ratio is close to one. Therefore, OPs in both *B. subtilis *and *E. coli *were well-conserved across many genomes, compared to the others. In contrast, conservation of NOPs drastically decreased along with the increasing conservation ratio. SOPs appear to be intermediate between OPs and NOPs. All the pairs of them were significantly different by a chi-squred test (p < 0.01). These results indicate that gene order is often corrupted at the regulatory boundary and corruption can occur even within an operon if some different TUs overlap.

### Co-expression levels of adjacent gene pairs

**Figure 4:**
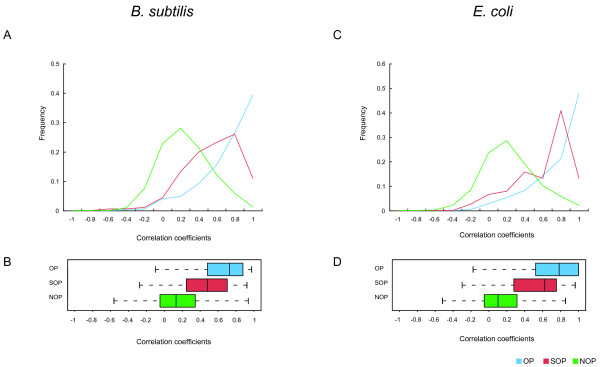
**Distributions of frequency of correlation coefficients of gene expression profiles between adjacent gene pairs**. The upper figures are the distribution of correlation coefficients (A and C) and the bottom figures are their box plots (B and D). Blue, red and green lines indicate OPs, SOPs and NOPs, respectively.

To investigate the differences in the gene expression of OPs, SOPs and NOPs, we measured the correlation with co-expression data calculated using several microarray data sets (see Materials and Methods). Figure 4 shows the distributions of the correlation coefficients of gene expression profiles between adjacent gene pairs and the boxplots for the three groups in *B. subtilis *and *E. coli*. The distribution of OPs shows that they have an obviously higher correlation than NOPs (the blue line in the parts A and C of Figure 4). This result agrees with past studies, and such clear differences have been used to predict operons [8,10]. Interestingly, SOPs also show high correlation, but not as clearly as OPs. All pairs of these three groups were significantly different (p < IE-7 for OP-NOP, SOP-NOP and OP-SOP, wilcoxon rank sum test). These differences are also clear in the box plots in Figure 4 and the median values in Table 2. Both the ranges of quartiles and the median values show that OPs and SOPs are differently distributed. In this study, we found that the co-expression levels of gene pairs within an operon can decrease due to the presence of regulatory elements in their intergenic region.

### Operons in biological pathways

#### Co-occurence on biological pathway maps

To determine the functional similarities at the level of biological pathway maps in the three groups of adjacent gene pairs, we measured the frequency of co-occurrence on pathway maps in KEGG, which contains information about metabolic and regulatory pathways and molecular complexes. KEGG has about 300 diagrams of molecular interactions or reactions. The number of times that both genes in an adjacent gene pair in *B. subtilis *and *E. coli *appear on the same and different KEGG pathway maps is shown in Table 2. If either gene of an adjacent gene pair was not mapped to a KEGG pathway, it was counted as being assigned to different maps. If both genes were not assigned to any maps, we ignored them. In particular, NOPs were dominated by gene pairs occurring in different pathway maps. Adjacent gene pairs in OPs frequently appeared on the same pathway maps, in contrast with NOPs (p < 1E-15, Fisher's exact test). Additionally, although only a small number of gene pairs in SOPs and NOPs co-occurred on the same pathways, SOPs significantly co-occured more often (p < 1E-6 for Fisher's exact test). Furthermore, OPs are co-occured less than expected in different pathway maps (p < 1E-7, Fisher's exact test). On the other hand, SOPs are not significantly different from NOPs using Fisher's exact test.

#### Co-occurence in functional categories

**Figure 5:**
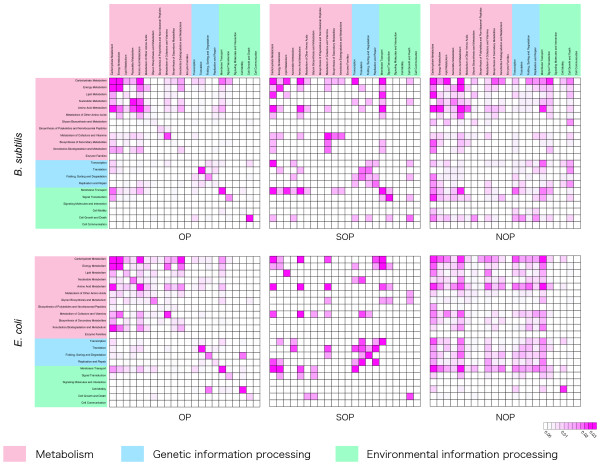
**Functional associations between adjacent gene pairs**. The upper figures indicate the functional associations in *B. subtilis *and the lower represent *E. coli*. The proportion of gene pairs that have the functional categories shown on the x and y axis is represented by a box with red color. When the color is deeper, it indicates the proportion is increasing.

To get a broader point of view than only the co-occurence on biological pathway maps, we measured the co-occuring frequency of these three groups of adjacent gene pairs on hierarchical categories of gene functions. We counted the number of gene pairs that shared the same category at the second level of the hierarchy (e.g. Carbohydrate metabolism). We used 22 categories that are related to prokaryotes. In Figure 5, each box represents the proportion of gene pairs that have the functional categories given on the x and y axis in *B. subtilis *(top) and *E. coli *(bottom). The deeper color indicates increasing frequency of gene pairs with the given functional categories. Additional file 1 includes the statistical analysis of these functional sharings using chi-square values. In OPs, the diagonal boxes are clearly represented by the deep red color. Statistical analysis reveals significant diagonal factors in functional sharing of OPs, even compared to Figure 5. Thus, gene pairs in OPs tend to have related functions. On the other hand, it was clear that almost all of the boxes as well as the diagonals in NOPs did not show high values. Particularly in statistical analysis they were almost similar as expected, so they did not share the related functions and can be randomly distributed. More gene pairs in SOPs were in related functional categories, compared to NOPs. In addition, gene pairs in the 'Genetic information processing' and 'Environmental information processing' groups were more likely to share functions in SOPs, compared to the other two groups. This trend was clearly shown in statistical analysis in Additional file 1. 'Transcription', 'Translation', 'Folding, Sorting and Degradation' and 'Replication and Repair' in 'Genetic information processing', and 'Membrane Transport', 'Signal Transduction' and 'Cell Growth and Death' in 'Environmental information processing' showed significant correlations. It seems to be an inherent property of SOPs that the degree of sharing between such categories and 'Metabolism' is also relatively frequent. We speculate that the functional bias of SOPs to these categories relates to the regulation of gene expression because SOPs are located on the boundary of the regulatory elements.

## Discussion

### Properties of operons from a genomic perspective

The intergenic regions were clearly shorter in OPs and SOPs than in NOPs (Figure 2). Genes co-transcribed as an operon are likely to be compactly arranged on the genome. It is suggested that short intergenic regions would help to allow efficient transcription. Interestingly, we found that the distributions of the intergenic regions of OPs and SOPs also appear to have different shapes (Figure 2). This observation suggests the possibility of the presence of regulatory elements such as internal promoters and internal terminators in the intergenic regions of SOPs. Actually, there are known cases where such regulatory elements cause variations in the length of transcriptional units. For example, the *sig*B and *res*ABCDE operons in *B. subtilis *have upstream and internal promoters, resulting in two TUs [22,23], and transcriptional terminations of operons such as the *bmr *and *bio *operons are also experimentally verified to be transcribed from the upstream promoter to the internal and external terminators, resulting in two different sizes of TUs [24,25]. The *sig*B operon consists of eight genes, *rsb*R-S-T-U-V-W-*sig*B-*rsb*X, and is transcribed from an upstream sigma A dependent promoter and from an internal heat-inducible sigma B dependent promoter [22]. The eight genes are usually co-transcribed by sigma A. When sigma B is activated in response to heat stress, it promotes transcription of the *sig*B regulon from the internal promoter, resulting in a shorter TU, *rsb*V-W-sigB-*rsb*X. The intergenic distance at the internal transcriptional boundary between *rsb*U and *rsb*V is 64 bp, whereas those of the others are 7, 6, 14, -1, -38 and 2, and their average is -1.7. Thus, the presence of regulatory elements seems to correspond to expanded intergenic regions. When the alternative transcripts are produced, most of them are caused by transcriptional regulatory elements located in the intergenic region at the boundary of the TU. Therefore, the longer intergenic regions of SOPs compared to OPs imply the presence of regulatory elements such as internal promoters and internal terminators. In addition, transcription can also be regulated by the presence of readthrough terminators which void specific termination signals, or by regulatory mechanisms such as riboswitches. Even if the specific promoters or terminators in a SOP region have not been identified, other transcriptional mechanisms may have an effect on the transcription.

### Properties of operons from transcriptomic perspective

According to our microarray expression analysis, OPs clearly showed high correlation in contrast to NOPs (Figure 4). It is quite reasonable that gene pairs within a TU are highly correlated. In addition, the correlations of OPs and SOPs also appear to be differently distributed according to the range of the quartiles (Figure 4). Hence, the gene expressions of these groups showed similar relationships to the intergenic distances. As shown in our results, the three groups differed in both genome organization and transcriptomic profiles. The differences would suggest different regulatory mechanisms of transcription and the functions of these genes in cellular processes.

#### Complicated operon structures

**Figure 6:**
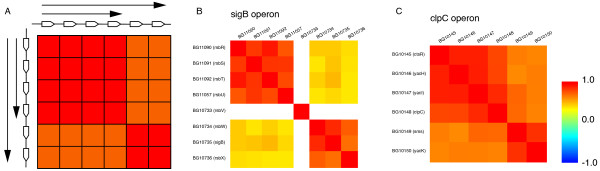
**Co-expression profile of operons: the schematic model of the operon diversity and its examples**. (A) Schematic model of a diverse operon. In this model, we assume that there is a longer TU and a shorter TU. Arrows indicate the region transcribed as a TU. Open boxes to the side and above the grid indicate genes. Each box in the grid indicates the level of correlation of gene expression as colors from blue to red correspond to the correlation coefficients. The gene pairs without the TU boundary should show strong correlation with vivid red color. On the other hand, gene pairs across the boundary should show a slightly weaker correlation with orange color. (B) The *sig*B operon. There are two promoters in the *sig*B operon. The first one is located in the upstream of the first gene of the operon, and second one is located in between fourth and fifth genes. Therefore, two different transcripts cause this pattern. (*rsb*V in this operon was not measured in the microarray experiments, so the region of this gene is not colored.) (C) The *clp*C operon. The transcription of this operon is basically promoted by sigma B, while the last two genes (*sms *and *yac*K) were reported to be also transcribed by sigma M.

Figure 6 illustrates the levels of co-expression of genes comprising an operon with possible overlapped TUs. Boxes colored from blue to red, corresponding to increasing values of the correlation coefficients, indicate the levels of co-expression. They are arranged in the order of genes on the genome. As a simple case, suppose that there is an operon with two different sized TUs (Figure 6A). The longer TU has an additional two genes compared to the shorter one but both start from the same gene. In this case, the gene pairs crossing the boundary between the shorter and longer TUs will be correlated, but the other gene pairs should be more strongly correlated (Figure 6A).

From a practical viewpoint, various situations may occur: (i) all genes within an operon have strong correlation with each other; (ii) there are internal terminators within an operon; (iii) there are internal promoters within an operon; (iv) there are other regulatory mechanisms such as readthrough terminators. For example, Figure 6B is the correlation matrix for the *sig*B operon described in the previous section (*rsb*V in this operon was not measured in the microarray experiments, so the region of this gene is not colored), and shows a similar pattern in the schematic model in Figure 6A. The image correctly suggests two different sized transcripts in the operon.

Figure 6C shows another example. The *clp*C operon in *B. subtilis *is transcribed as a six gene operon including *cts*R, *yac*H, *yac*I, *clp*C, *sms *and *yac*K [31]. This operon is related to the control of competence and survival under various stress conditions. Two promoters are mapped upstream of this operon. One is a sigma A-like promoter and the other is dependent on sigma B [22,32]. In addition, it was reported that the last two genes of this operon might be also a part of operons regulated by sigma M [22,33]. As suggested by the image, these reports imply that there are longer transcripts comprised of six genes and another transcript including just the last two genes.

### Functional relationships of operon structures

Gene clusters obtained by comparative genomics are likely to be operons, and they also tend to cluster on metabolic pathways [1-3]. We measured the relationship between OPs, SOPs and NOPs with KEGG biological pathway maps. As shown in Table 2, gene pairs in OPs tend to appear in the same KEGG pathway maps. Therefore, genes within an operon are more often closely located on metabolic pathways. On the other hand, almost all gene pairs in NOPs occurred on different pathway maps (Table 2). This suggests that the boundaries between operons are clearly split according to functional relations. 11 NOPs in *B. subtilis *were, however, mapped to the same pathways. For example, *roc*A constitutes an operon with *roc*B and *roc*C, among four consecutively located genes: genes *roc*G, *roc*A, *roc*B and *roc*C. This operon is not coregulated with *roc*G due to the presence of a specific enhancer located between *roc*G and *roc*A. So the gene pair, *roc*G-*roc*A, is regarded as a NOP, while both genes belong to glutamate metabolism. This gene pair is also assigned to other pathways: nitrogen metabolism (*roc*G) and arginine and proline metabolism (*roc*A). The other NOPs that appear on the same map and also appear on alternative pathway maps are, *his*C-*trp*A (phenylalanine, tyrosine and tryptophan biosynthesis), *spo*VD-*mur*E (peptidoglycan biosynthesis), *trp*E-*aro*H (phenylalanine, tyrosine and tryptophan biosynthesis), and *men*C-*men*E (ubiquinone biosynthesis). On the other hand, the remaining six pairs, *hxl*A-*hxl*B (pentose and glucuronate interconversions), *yfl*S-*yfl*R (two-component system), *puc*E-*puc*H (purine metabolism), *yly*B-*pyr*R (pyrimidine metabolism), *pbp*B-*spo*VD (peptidoglycan biosynthesis), and *yvr*P-*fhu*C (ABC transporters), are assigned to only the same pathway map. In *E. coli*, 35 NOPs were mapped to the same pathways. Of these pairs, 18 NOPs are assigned to the same map and the rest of them are assigned to multiple maps. These functionally related NOPs can be regarded as gene pairs similar to SOPs in the sense that the gene order indicates their operon structure, but they are not directly co-regulated. In addition, from the comparative analysis, NOPs were either not adjacently conserved or lost in the other genomes. If an adjacent gene pair is not co-regulated in the same manner even if they are related to the same process, their gene order would be less conserved. Thus, a small fraction of NOPs are very similar to SOPs. Furthermore, the fact that SOPs occur more on different pathway maps is a similar tendency to NOPs, compared with those of OPs. Therefore, SOPs and NOPs may be relatively close in functional relationships. This implies that SOPs as well as NOPs may also play a role in the functional boundaries that produce a suitable set of proteins in a certain environment by alternative promoters or terminators, although such functional differences of SOPs are not as clear as those of NOPs.

Because almost half of all OPs were distributed in different biological pathway maps in Table 2, we can speculate that these genes in the same operon can have diverse functions. However, distribution of broader functional categories in Figure 5 and Additional file 1 (statistical distribution based on chi-square value) clearly show that the functional relationships of OPs are quite significant. The map-based analysis may be too specific to see the general trends in functional relationships of OPs. It is also interesting that gene pairs in SOPs share more functions related to genetic information and environmental responses such as transcription, translation and signal transduction, compared to the other two groups, OPs, and NOPs (Figure 5 and Additional file 1). This suggests that such functions are associated with the regulatory changes causing the transcription of alternative transcriptional units. As described in the previous section, it has been observed that some environmental factors trigger transcriptional unit changes. Therefore, it is understandable that some SOPs have a bias to these functions.

In addition, we have shown that SOPs are less conserved than OPs from the comparison of about 200 prokaryotes (Figure 3). Although it has been reported that operon structures are not stable throughout the evolutionary process [34], our result suggests that the collapse of operon structures has occurred frequently at the region of regulatory boundaries including SOPs and, in particular, NOPs. Recently, Price *et al*. have reported that, during operon evolution, a new gene is more likely to append to the end of a pre-existing operon and it is often a functionally unrelated gene [35]. The facts found by them suggest that these appending genes may be the origin of SOPs. Therefore, these SOPs and functionally related NOPs described above would play an important role in the evolution of operons. Moreover, it has been observed that even if genes found in an operon in a given genome are split in another genome, they can be co-regulated by a single regulon in the given genome [17,36]. Therefore, we suggest that complicated operon structure and regulon structures in different organisms, although they have different regulatory mechanisms, are evolutionary associated with each other. To clarify these relationships, highly reliable operon and regulon predictions are required. However, the intricate transcriptional regulation we have shown here makes this difficult. Our on-going project is to improve such predictions using the operon features that we have shown here and to uncover gene regulatory mechanisms across a variety of genomes. In this study, we found that there are the interesting differences among OPs, SOPs and NOPs. However, it still remain that higher statistical analysis could solve the inter-dependence among genomic, transcriptomic and functional features of gene pairs.

## Conclusion

We classified adjacent gene pairs into three groups (OP, SOP and NOP) according to the levels of gene co-regulation in operon structures including substructures such as alternative TUs. Consequently, we found that the levels of gene co-regulation are correlated with genome organization, gene expression profiles and conservation across genomes. Interestingly, we found that functional associations of SOPs are often observed in the environmental and genetic information processing functional classes in KEGG. This is the first report of these relationships between operon organization and transcriptional units including substructures in operons, and we suggest that the strength of gene associations in an operon play an important role in environmental accommodation and in evolution of gene order across genomes.

## Methods

### Genomic data

The genome information for *B. subtilis *and *E. coli *was prepared from the KEGG GENES database [37,38]. By using the information of the positions of genes, we classified adjacent gene pairs into the following three groups (Figure 1): OPs, SOPs and NOPs. We also calculated the intergenic distances between all the gene pairs. The distance was defined as the number of bases separating adjacent gene pairs on the same strand on the genome. If they have an overlapped region, the distance is negative.

### Operon data

We have obtained the information on TUs from ODB and RegulonDB. A summary of the known TUs in *B. subtilis *and *E. coli *is shown in Table 1. ORFs in Table 1 mean the total number of genes organizing the known TUs in ODB and RegulonDB. We obtained 688 TUs in *B. subtilis *and 396 TUs in *E. coli *from ODB and 693 TUs in *E. coli *from RegulonDB. If two TUs overlap but contain different sets of genes, we regarded these units as different TUs. These overlapped TUs share same genes with different TUs.

### Evaluation of conservation of adjacent gene pairs

We obtained the genomic data of 185 prokaryotic organisms from KEGG [37,38]. We summarize the organisms used in this comparative analysis in Additional file 2. To identify orthologs, we used OC [37,38], which is an ortholog clustering using the results of homology searches done by the Smith-Waterman algorithm [39,40]. In each adjacent gene pair, we counted the number of the gene pairs that the orthologs to both genes are on the other genomes. When both genes in a gene pair in a given genome are conserved in other genomes, the ratio is defined by dividing the number of genomes in which they are adjacently conserved by the number of total genomes in which they are conserved. In each measurement, we removed organisms closely related to *B. subtilis *and *E. coli *in the same taxonomic group, which are defined in KEGG.

### Similarity of gene expression profiles

We used 150 microarray experiments for *B. subtilis *performed under 10 different experimental growth conditions in BSORF [41] (Additional file 3). Gene expression intensities were obtained by subtracting background intensities [42-45]. If the intensity was less than the standard deviation of the backgrounds, it was treated as a missing value. A ratio of expression intensity was obtained by dividing the target intensity by the control intensity [42-45], which was transformed into a logarithm of base 2. Normalization was carried out by subtracting the median value in each experiment [45,46]. In addition, 140 microarray experiments for *E. coli *from Gene Expression Omnibus [47] (Additional file 4) with lowess normalization were collected. We then calculated the Pearson's correlation coefficients between all gene expression profiles.

### Evaluation of adjacent gene pairs in biological pathways

We obtained the KEGG PATHWAY information from the GenomeNet database [37,38]. KEGG PATHWAY is a knowledge base for molecular interaction networks, including metabolic pathways, regulatory pathways and molecular complexes. KEGG has about 300 diagrams of molecular interactions or reactions. In this study, 127 biological pathway maps for *B. subtilis *and 121 maps for *E. coli *were used. We extracted the set of the genes that belong to each pathway. We counted the genes that matched with those in OPs, SOPs and NOPs. In each group, we counted the number of genes that appeared in the same and different pathway maps. In addition, these maps are classified into hierarchical categories. We used 22 categories at the second level of the hierarchy (e.g. Carbohydrate metabolism) that are related to prokaryotes, in which 12 are included in metabolisms, 4 in genetic information processing and 6 in environmental information processing. We counted the number of the corresponding gene pairs for each category pair.

## Authors' contributions

SO performed all of the analyses and wrote the manuscript. KK and NO were involved in the designing and running of the microarray expriments. SK, MK and SG participated in design and coordination of the study. All authors read and approved the final manuscript.

## Supplementary Material

Additional File 1 **Statistical analysis of functional association**. Statistical analysis of the functional sharings is performed based on chi-square values. Click here for file

Additional File 2 **Species used in this study**. We summarize the species names used in the comparative analysis of 185 prokaryotic organisms from KEGG. Click here for file

Additional File 3 **Microarray data in *B. subtilis***. 150 microarray experiments for *B. subtilis *under 10 different experimental growth conditions. Click here for file

Additional File 4 **Microarray data in *E. coli***. 140 microarray experiments for *E. coli *from Gene Expression Omnibus. Click here for file

## Figures and Tables

**Table 1:** The information of known TUs in *B. subtilis *and *E. coli*

	*B. subtilis*	*E. coli*
Number of TUs	688	754
Number of overlapped TUs	334	211
Number of ORFs	1163	1545

**Table 2:** Statistics of OPs, SOPs and NOPs in *B. subtilis *and *E. coli*

	*B. subtilis*			*E. coli*		
	
	OP	SOP	NOP	OP	SOP	NOP
Number of pairs	527	182	663	811	112	1088
Median of intergenic distances (bp)	17	72	368	9	43	457
Median of correlation coefficients of co-expression	0.73	0.48	0.13	0.78	0.62	0.10
Number of co-occurrence on the same pathway maps	192	22	11	367	20	35
Number of co-occurrence on the different pathway maps	113	57	314	201	57	581
