# Supplementary material for: Characterization of relationships between transcriptional units and operon structures in Bacillus subtilis and Escherichia coli
Source: BMC Genomics. 2007 Feb 13;8:48. doi: 10.1186/1471-2164-8-48 (PMC1808063; doi:10.1186/1471-2164-8-48)
Supplement: Additional File 1 — Statistical analysis of functional association. Statistical analysis of the functional sharings is performed based on chi-square values. [file 1471-2164-8-48-S1.pdf]

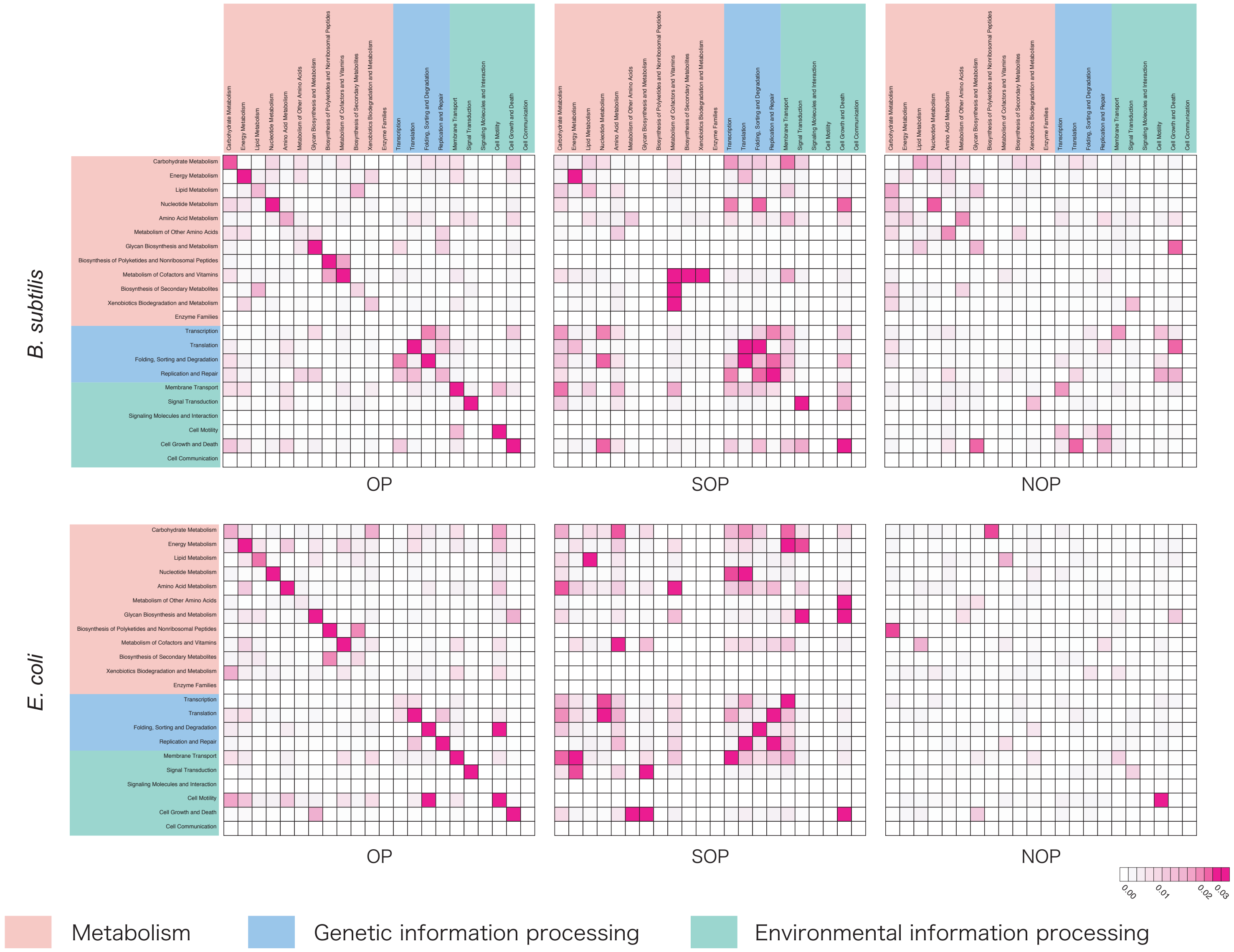

Statistical analysis of functional associations.

We performed a statistical analysis of Figure 5. We estimated the relevance of observed values compared to expected values from the marginal distributions. The expected value within each box was simply the product of the row total and column total (in marginal distributions) divided by the overall sample number. The relevance of the box was the squared difference between the observed and expected values divided by the expected value (as in a chi-squared test). This value becomes high, when the observed value is greatly different from the expected. In this figure, each box represents such significant value with red color. To compare difference among OPs, SOPs and NOPs, the values were divided by the total number of them in each groups.
